# Outcomes of Patients Living with HIV Hospitalized due to COVID-19: A 3-Year Nationwide Study (2020–2022)

**DOI:** 10.1007/s10461-024-04394-z

**Published:** 2024-07-04

**Authors:** Rafael Garcia-Carretero, Oscar Vazquez-Gomez, Belen Rodriguez-Maya, Ruth Gil-Prieto, Angel Gil-de-Miguel

**Affiliations:** 1 Department of Internal Medicine, Mostoles University Hospital, Rey Juan Carlos University (Madrid), Madrid, Spain; 2 Department of Preventive Medicine and Public Health, Rey Juan Carlos University (Madrid), Madrid, Spain

**Keywords:** COVID.19, Mortality, HIV, Population-based

## Abstract

Scientific reports on the association between human immunodeficiency virus (HIV) in patients with COVID-19 and mortality have not been in agreement. In this nationwide study, we described and analyzed the demographic and clinical characteristics of people living with HIV (PLWH) and established that HIV infection is a risk factor for mortality in patients hospitalized due to COVID-19. We collected data from the National Hospital Data Information System at Hospitalization between 2020 and 2022. We included patients admitted to the hospital with a diagnosis of COVID-19. We established a cohort of patients with PLWH and compared them to patients without HIV (non-PLWH). For multivariate analyses, we performed binary logistic regression, using mortality as the dependent variable. To improve the interpretability of the results we also applied penalized regression and random forest, two well-known machine-learning algorithms. A broad range of comorbidities, as well as sex and age data, were included in the final model as adjusted estimators. Our data of 1,188,160 patients included 6,973 PLWH. The estimated hospitalization rate in this set was between 1.43% and 1.70%, while the rate among the general population was 0.83%. Among patients with COVID-19, HIV infection was a risk factor for mortality with an odds ratio (OR) of 1.25 (95% CI, 1.14–1.37, *p* < 0.001). PLWH are more likely to be hospitalized due to COVID-19 than are non-PLWH. PLWH are 25% more likely to die due to COVID-19 than non-PLWH. Our results highlight that PLWH should be considered a population at risk for both hospitalization and mortality.

## Introduction

The coronavirus disease 2019 (COVID-19), caused by the SARS-CoV-2 virus, imposed a great burden of illness worldwide, from both socioeconomic and healthcare points of view. As of July 5, 2023, a total of 13,914,811 confirmed cases of COVID-19 had been reported in Spain, with 682,216 hospitalizations and 122,057 deaths [1]. Some comorbidities are considered risk factors for adverse effects and mortality. Chronic conditions, such as diabetes, hypertension, obesity, immunosuppression, malignancies, and HIV infection, have been associated with worse outcomes in COVID-19 [2].

People living with HIV (PLWH) may be proportionally more affected by infection with SARS-CoV-2 than people without HIV (non-PLWH), although conflicting results have been reported [3]. If coinfection with HIV and SARS-CoV-2 puts patients at high risk for mortality, health systems should engage in more aggressive preventive measures and therapeutic efforts to avoid adverse outcomes in PLWH when infected by SARS-CoV-2.

In this study, we analyzed the impact of coinfection with HIV and SARS-CoV-2 in PLWH. We describe the incidence of hospitalization among PLWH and related outcomes in terms of comorbidities, severity, and mortality.

## Methods

### Study Design

We conducted a retrospective, population-based study using data drawn from the Spanish National Hospital Data Information System at Hospitalization (MBDS-H), a valuable database for epidemiological analyses of included conditions created by the Spanish Ministry of Health. The MBDS-H is an administrative registry of discharge reports. Nearly 95% of hospitals in Spain, both public and private, are covered by the database. It is estimated that 97% of all discharge reports are registered in this database. The data are exclusively drawn from hospital discharges, including information on age, sex, date of admission/discharge, type of hospital, place of residence, and diagnoses. MBDS-H includes diseases encoded using the 10th Clinical Revision of the International Classification of Diseases (ICD-10-CM). A new dataset is generated in January of each year. However, due to the high volume of data, the data only become available after a delay of 1 year. The Spanish Ministry of Health provided us with data up to December 31, 2022.

### Data Collection

We used data from populations covered by hospitals included in the MBDS-H information system, as noted. We were provided with the microdata extracted from the MBDS-H from the Ministry of Health between 2020 and 2022 using the code for COVID-19 (U07.1) in any diagnostic position. That is, we collected data for patients presenting with a diagnosis of COVID-19 from January 1, 2020, to December 31, 2022. For HIV infection, we used the codes Z21 and B20 to B24. No data on treatment or immunovirological status were provided. For each hospitalized patient, we collected data on age, sex, dates of admission and discharge, ICU admission, and type of discharge. Main and secondary diagnoses were also gathered to identify HIV infection, diabetes, hypertension, and other chronic conditions. Patients who had incomplete data regarding ICU admission, mortality, length of hospitalization, or diagnosed conditions of interest were excluded. No names or personal identifying details were recorded. Data were anonymized and de-identified to ensure patients’ privacy.

### Definition of Waves

We categorized the pandemic into waves based on the classification of the Epidemiological National Surveillance Net study, which exclusively used data from Spain. The observation periods were split into outbreaks based on the 14-day cumulative incidence and on a turning point for each wave, such that every turning point indicated the end of one wave and the beginning of the next [4].

### Univariate Analysis

We performed descriptive and correlational analyses. We used means or medians with continuous variables as appropriate, as well as percentages with categorical variables. Average hospital length of stay is defined as the total number of days of stay, divided by the total number of hospitalizations. Mortality and the need for ICU admissions are considered clinical severity criteria. Deaths and ICU admissions, as numerators, are divided by the total number of hospitalizations to calculate the mortality rate and ICU admission rate, respectively. Both parameters are expressed as percentages. The chi-square test and the Wilcoxon signed-rank test were performed as tests of independence when appropriate.

### Multivariate Analyses

Logistic regression was used to analyze mortality in our cohort and hence to estimate the impact of the included variables. We used a combination of a classical approach (with binary logistic regression) and a machine learning approach (with penalized logistic regression) to calculate beta coefficients for variables as well as odds ratios (ORs). Binary logistic regression is the most frequently used statistical approach in biomedical sciences with binary outcomes, i.e., yes/no. Logistic regression is simple and straightforward, and it provides easy interpretation of the effects of explanatory variables on response variables. However, a model may have too many features selected as explanatory variables, making it too complex for use. The rationale for the use of machine learning at this stage was that it allowed us to select a set of features, that is, a parsimonious model, without loss of accuracy or reliability.

As noted, our machine learning approach adopted logistic regression with L1-penalized regularization. This approach is also known as the least absolute shrinkage and selection operator (LASSO) [5, 6]. It discards variables that do not contribute to the fit of the final model. It forces beta coefficients to a range from very small values to exactly zero. All beta coefficients shrink, but those with weak effects are dropped. We used cross-validation to internally validate the LASSO algorithm. We plotted the average model evaluation scores to select the set of variables that maximized the model’s predictive accuracy. Penalization is determined by the lambda value, which was used to select the subset of variables. LASSO is recommended by the Transparent Reporting of a Multivariable Prediction Model for Individual Prognosis or Diagnosis checklist for developing and validating risk and diagnostic models [7].

### Interpretability of Results

Once a subset of features was selected, the beta coefficients were obtained, and the ORs were calculated, we used random forest (RF), another machine learning algorithm based on decision trees, to better interpret the results. This generated many independent decision trees, which then were combined to obtain a single output [8, 9]. RF allowed the model to be interpreted at a global scale.

For all tests, the level of statistical significance was set at *P* < 0.05. We used R language version 4.3.2 (Vienna, Austria) running on a Debian 12 GNU/Linux workstation for both standard and machine learning-based analyses.

## Results

We assessed data on 1,188,160 hospitalized patients, including 6,973 patients living with HIV (0.58% of all hospitalized patients) between 2020 and 2022. The main characteristics are presented in Table 1. Men among PLWH were more likely to be hospitalized than men among non-PLWH. PLWH were also significantly younger than non-PLWH. They also had a lower prevalence of diabetes, hypertension, coronary disease, heart failure, and other cardiovascular risk factors. However, chronic liver disease, malignancy, and chronic pulmonary diseases were significantly more common in PLWH.

By December 31, 2021, it was estimated that there were between 136.436 and 162.307 PLWH in Spain. That is, the prevalence of HIV in Spain was between 0.28% and 0.31%. The rate of hospitalization of patients among the general population was 0.83% per year between 2020 and 2022. Regarding PLWH, the ratio of hospitalized patients was between 1.43% and 1.70%, that is, greater than the general population.

**Table 1:** Baseline features of our cohort and univariate analyses

	Total	HIV infection		Test statistic	*P* value
		PLWH	No PLWH		
**Patients**	1,188,160	6,973	1,181,187	NA	NA
**Sex (men)**	54.9%	75.3%	54.8%	X^2^ = 1.18 × 10^3^	0.001
**Age (IQR)**	73 (27)	54 (12)	73 (27)	W = 6.28 × 10^9^	0.001
**Hospital length of stay in days (IQR)**	10.9 (8)	12.1 ( 9)	10.9 (8)	W = 5.9 × 10^0^	0.002
**ICU admissions (rate, %)**	100,578 (8.5%)	745 (10.7%)	99,833 (8.5%)	X^2^ = 5.51 × 10^1^	0.001
**ICU length of stay in days (IQR)**	8 (20.2)	7 (17.7)	8 (20.2)	W = 3.93 × 10^7^	0.006
**Deaths (rate, %)**	153,144 (12.9%)	540 (7.7%)	152,604 (12.9%)	X^2^ = 2.04 × 10^3^	0.001
**Comorbidities**					
Diabetes	24.4%	11.4%	24.5%	X^2^ = 6.46 × 10^2^	0.001
Hypertension	31.9%	16.7%	32%	X^2^ = 7.44 × 10^2^	0.001
Coronary disease	9.7%	7%	9.7%	X^2^ = 5.97 × 10^1^	0.001
Heart failure	17%	4.9%	17.1%	X^2^ = 7.26 × 10^2^	0.001
Dementia	6.5%	1.1%	6.6%	X^2^ = 3.42 × 10^2^	0.001
Chronic kidney disease	15.6%	9.7%	15.6%	X^2^ = 1.83 × 10^2^	0.001
Chronic liver disease	0.6%	5.8%	0.6%	X^2^ = 3.26 × 10^3^	0.001
Malignancy	10.1%	12.1%	10.1%	X^2^ = 3.09 × 10^1^	0.001
Obesity	11.8%	5.7%	11.9%	X^2^ = 2.53 × 10^2^	0.001
Chronic pulmonary disease	14.8%	26.8%	14.7%	X^2^ = 8.07 × 10^2^	0.001
Cerebrovascular disease	1.4%	0.6%	1.4%	X^2^ = 3.30 × 10^1^	0.001
**Coinfection**					
HBV	0.3%	4.2%	0.3%	X^2^ = 2.99 × 10^3^	0.001
HCV	0.7%	25.5%	0.6%	X^2^ = 5.77 × 10^4^	0.001
HTLV-I/II	0%	0%	0%	NA	NA

PLWH: people living with HIV. HBV: Hepatitis B virus. HCV: Hepatitis C virus. HTLV-I/II: Human T-lymphotropic virus types I and II. NA: non-applicable. ICU: intensive care unit. IQR: interquartile range. W: Wilcoxon signed-rank test. X^2^: Chi-square test

Overall, the prevalence of HIV and hepatitis B virus (HBV) coinfection was 0.7%, but we found that among PLWH the prevalence was 4.2%. Regarding hepatitis C virus, the prevalence among PLWH was even higher (25.5%). We did not found infection caused by human T-lymphotropic virus types I and II.

**Fig. 1:**
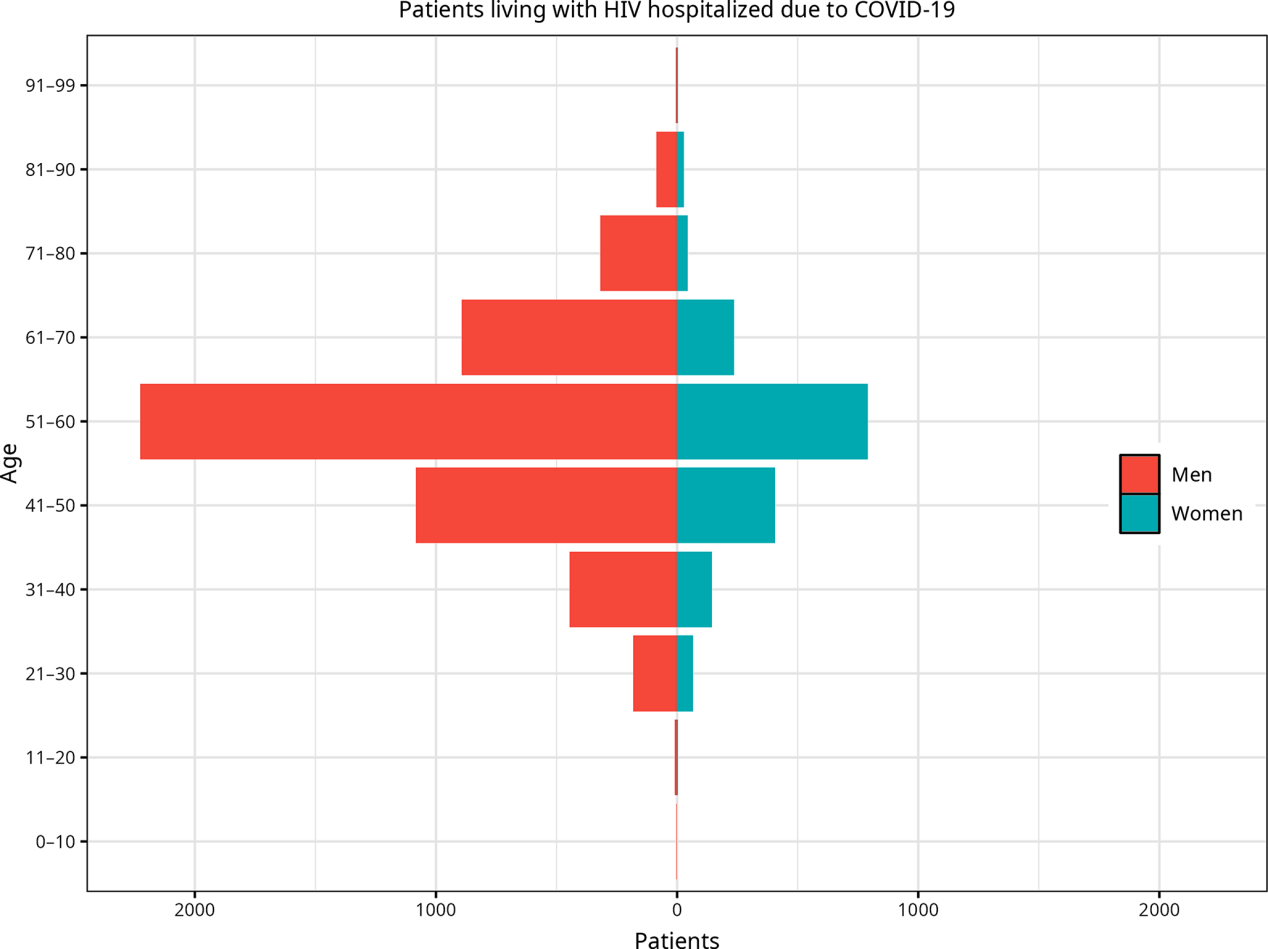
Population pyramid by age range among hospitalized people living with HIV between 2020 and 2022

Due to the predominance of men among PLWH, we decided to plot the relationship between age and sex in a population pyramid (Fig. 1) and to show the main characteristics related to COVID-19 in Table 2. It can be seen that the mortality rate in men was higher than it was in women (8.5% vs. 5.6%, X^2^ = 25.7, *p* < 0.001). In addition, cardiovascular risk factors, such as diabetes and coronary diseases, were more prevalent in men. Malignancy was also more prevalent in male PLWH. However, women tended to be more obese and to have a higher prevalence of chronic pulmonary disease. There were no differences regarding hypertension, heart failure, dementia, chronic kidney disease, chronic liver disease, or cerebrovascular disease.

**Fig. 2:**
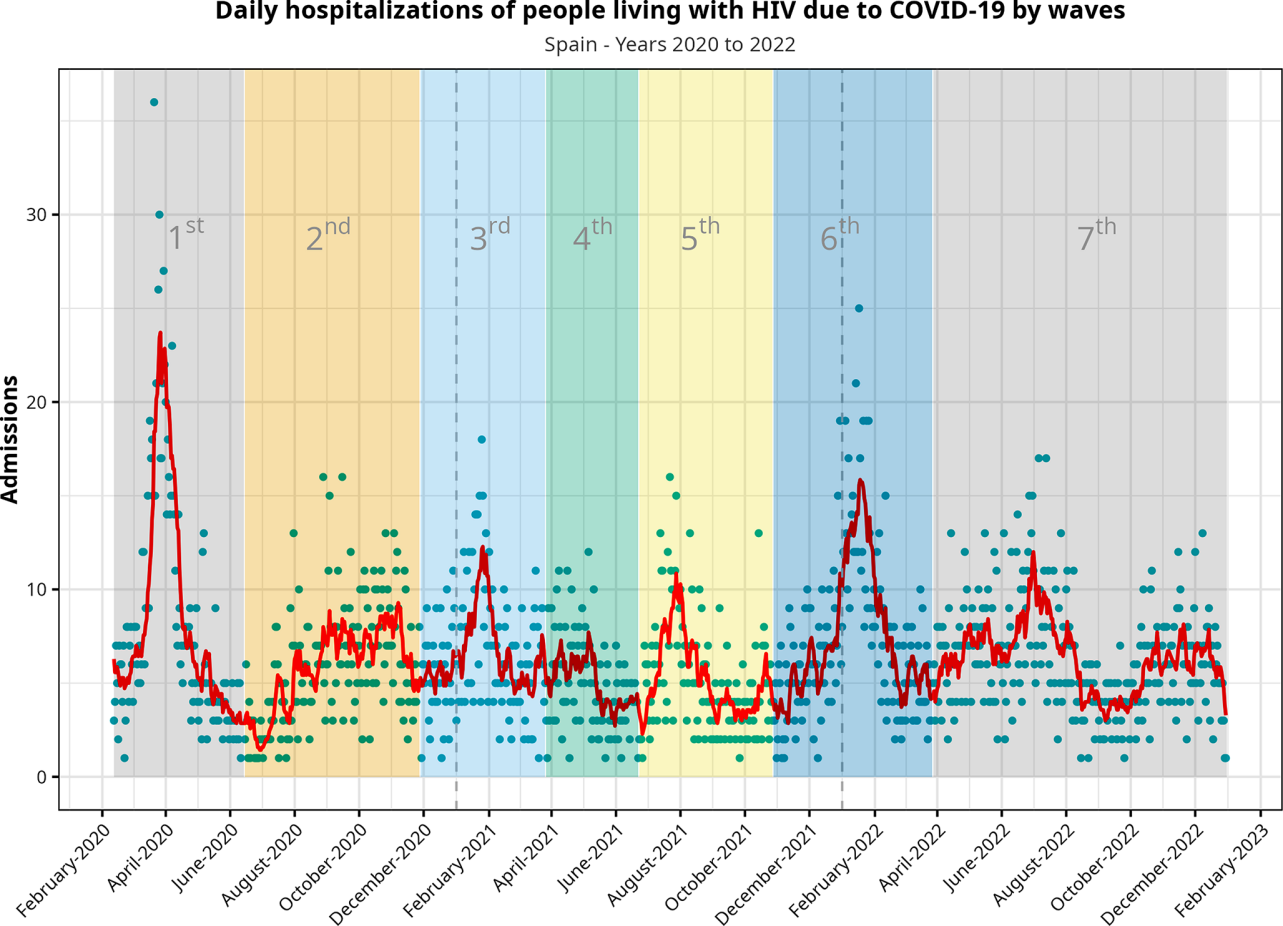
Evolution of the pandemic during the observation period regarding people living with HIV

Figure 2; Table 3 present the evolution of the pandemic among PLWH, splitting the observation period into epidemiological waves. The first wave included 1,303 hospitalizations, and this number steadily dropped until the fourth and fifth waves. More men were admitted than women, with no changes in the distribution along the pandemic. The median age was 54 years, with almost no changes for the entire period. While the mortality rate was 7.7% globally, we observed a decreasing trend from the third wave onward.

**Table 2:** Differences between men and women in the hospitalized Spanish population living with HIV

	Men	Women	Test statistic	*P* value
**Patients**	5,253	1,720	NA	NA
**Age (IQR)**	55 (12)	53 (12)	W = 4.92 × 10^6^	0.062
**Hospital length of stay in days (IQR)**	12.3 (9)	11.7 (8)	W = 1.10 × 10^0^	0.274
**ICU admissions (rate, %)**	586 (11.2%)	159 (9.2%)	X^2^ = 5.18 × 10^0^	0.073
**ICU length of stay in days (IQR)**	8 (18.1)	6 (16.1)	W = 5.17 × 10^4^	0.031
**Deaths (rate, %)**	444 (8.5%)	96 (5.6%)	X^2^ = 2.57 × 10^1^	0.001
**Comorbidities**				
Diabetes	12.3%	8.7%	X^2^ = 1.68 × 10^1^	0.001
Hypertension	17%	15.9%	X^2^ = 1.18 × 10^0^	0.289
Coronary disease	8.1%	3.5%	X^2^ = 4.15 × 10^1^	0.001
Heart failure	5%	4.5%	X^2^ = 6.70 × 10^-1^	0.43
Dementia	1.1%	1.1%	X^2^ = 2.00 × 10^-2^	0.894
Chronic kidney disease	10%	8.8%	X^2^ = 1.92 × 10^0^	0.17
Chronic liver disease	5.8%	5.8%	X^2^ = 0.0 × 10^0^	1
Malignancy	13.1%	9%	X^2^ = 2.09 × 10^1^	0.001
Obesity	4.8%	8.4%	X^2^ = 3.22 × 10^1^	0.001
Chronic pulmonary disease	25.7%	30.2%	X^2^ = 1.35 × 10^1^	0.001
Cerebrovascular disease	0.6%	0.5%	X^2^ = 5.90 × 10^-1^	0.495
**Coinfection**				
HBV	4.4%	3.4%	X^2^ = 3.55 × 10^0^	0.55
HCV	25.6%	25.1%	X^2^ = 1.90 × 10^-1^	0.676
HTLV-I/II	0%	0%	NA	NA

HBV: Hepatitis B virus. HCV: Hepatitis C virus. HTLV-I/II: Human T-lymphotropic virus types I and II. NA: non-applicable. ICU: Intensive care unit. IQR: interquartile range. W: Wilcoxon signed-rank test. X^2^: Chi-square test

**Table 3:** Clinical characteristics of the hospitalized population living with HIV (2020–2021), by epidemic wave

	Total	First	Second	Third	Fourth	Fifth	Sixth	Seventh
Patients	6,973	1,303	1,004	649	491	598	1,181	1,747
Sex (men)	75.3%	76.7%	75.5%	78.6%	76.2%	74.9%	74.2%	73.7%
Age (IQR)	54 (12)	53 (11)	53 (13)	55 (12)	56 (11)	53 (16)	55 (12)	55 (12)
Hospital length of stay in days (IQR)	7 (9)	11.1 (8)	7 (10)	13.8 (9)	8 (9)	12.5 (9)	7 (9)	7 (9)
ICU admissions (rate, %)	745 (10.7%)	118 (9.1%)	101 (10.1%)	85 (13.1%)	56 (11.4%)	79 (13.2%)	130 (11%)	176 (10.1%)
Deaths (rate, %)	540 (7.7%)	117 (9%)	71 (7.1%)	65 (10%)	36 (7.3%)	47 (7.9%)	91 (7.7%)	113 (6.5%)

ICU: Intensive care unit. IQR: Interquartile range

The first multivariate analyses were performed with all patients included. Table 4 presents the results. Mortality was used as the dependent variable. We included coinfection with SARS-CoV-2 and HIV as an independent variable, alongside sex, age, and the remaining comorbidities in the final model of logistic regression. HIV infection was a risk factor for hospitalizations due to COVID-19, that is, a patient had a 25% greater chance if a PLWH than a non-PLWH to be hospitalized for COVID-19.

**Table 4:** Multivariate analysis using logistic regression with mortality as a dependent variable. All patients were included in the analysis

	OR	95%CI	*P* value
**HIV infection**	1.25	1.14–1.37	0.001
**Sex (man)**	1.35	1.34–1.37	0.001
**Age**	1.05	1.05–1.05	0.001
**Comorbidities**			
Malignancy	2.21	2.18–2.25	0.001
Diabetes	1.01	1.00–1.02	0.12
Coronary disease	1.09	1.07–1.11	0.001
Heart failure	1.07	1.05–1.08	0.001
Hypertension	0.93	0.92–0.95	0.001
Obesity	0.96	0.94–0.98	0.001
Dementia	1.24	1.22–1.27	0.001
Cerebrovascular disease	2.07	1.99–2.14	0.001
Chronic liver disease	1.59	1.5–1.7	0.001
Chronic kidney disease	1.14	1.12–1.16	0.001
Chronic pulmonary disease	0.72	0.71–0.73	0.001
**Coinfection**			
HBV	0.95	0.85–1.05	0.291
HCV	1.06	0.99–1.13	0.111

CI: Confidence Interval. OR: Odds Ratio. HBV: Hepatitis B virus. HCV: Hepatitis C virus

**Fig. 3:**
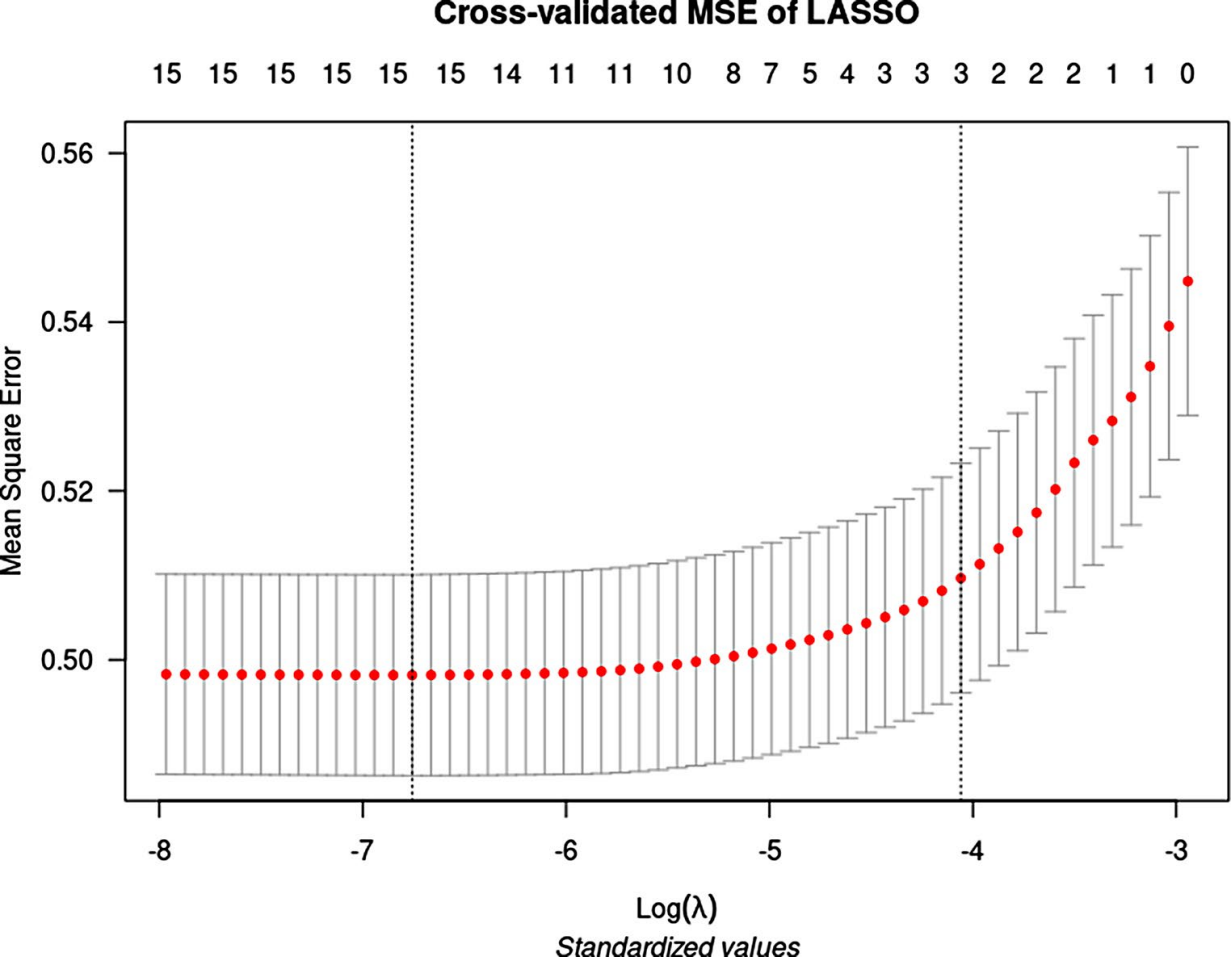
Lambda values for penalized regression (LASSO). Lambda values represent the penalization of the beta coefficients. Vertical lines represent the range of lambda for which accuracy is not adversely affected. Numbers across the top of the plot represent the number of variables in the model when a certain value of lambda was used (15 versus 3 features). We chose the lambda values that received the minimum number of features without losing accuracy (determined according to mean square error)

The results of the multivariate analysis of the data for PLWH using binary logistic regression are shown in Table 5. Sex, age, malignancies, heart failure, hypertension, and chronic liver disease were the main risk factors for a PLWH to be admitted to a hospital. To identify the most relevant variables, we also performed penalized logistic regression for mortality. Compared to the standard binary logistic regression, the list of variables was small, as LASSO dropped non-relevant variables as the model was being fitted. LASSO only identified age, malignancies, and heart failure as the most relevant variables associated with the risk of death. LASSO only provides beta coefficients so that ORs can be calculated, it does not report confidence intervals. In addition, these ORs tend to be lower than those in standard logistic regression, due to the characteristics of the algorithm. LASSO proposed a more constrained model using a lambda value that chose only three variables (Fig. 3).

**Table 5:** Multivariate analysis using logistic regression and penalized logistic regression among PLWH only, using mortality as a dependent variable

	Binary logistic regression		LASSO (penalized regression)	
	OR (95%CI)	*P* value	Beta coefficient	OR
Sex (man)	1.35 (1.07–1.72)	0.01	.	.
Age	1.04 (1.03–1.05)	0.001	0.01447651	1.01
Malignancy	4.21 (3.44–5.15)	0.001	1.03839103	2.82
Heart failure	2.11 (1.53–2.89)	0.001	0.08649972	1.09
Hypertension	0.72 (0.56–0.93)	0.01	.	.
Obesity	1.44 (0.99–2.06)	0.05	.	.
Dementia	0.41 (0.1–1.12)	0.13	.	.
Cerebrovascular disease	2.2 (0.9–4.81)	0.06	.	.
Chronic liver disease	1.51 (1.07–2.08)	0.01	.	.
Chronic pulmonary disease	0.65 (0.52–0.81)	0.001	.	.

OR: odds ratio. CI: confidence interval. LASSO: least absolute shrinkage and selection operator

**Fig. 4:**
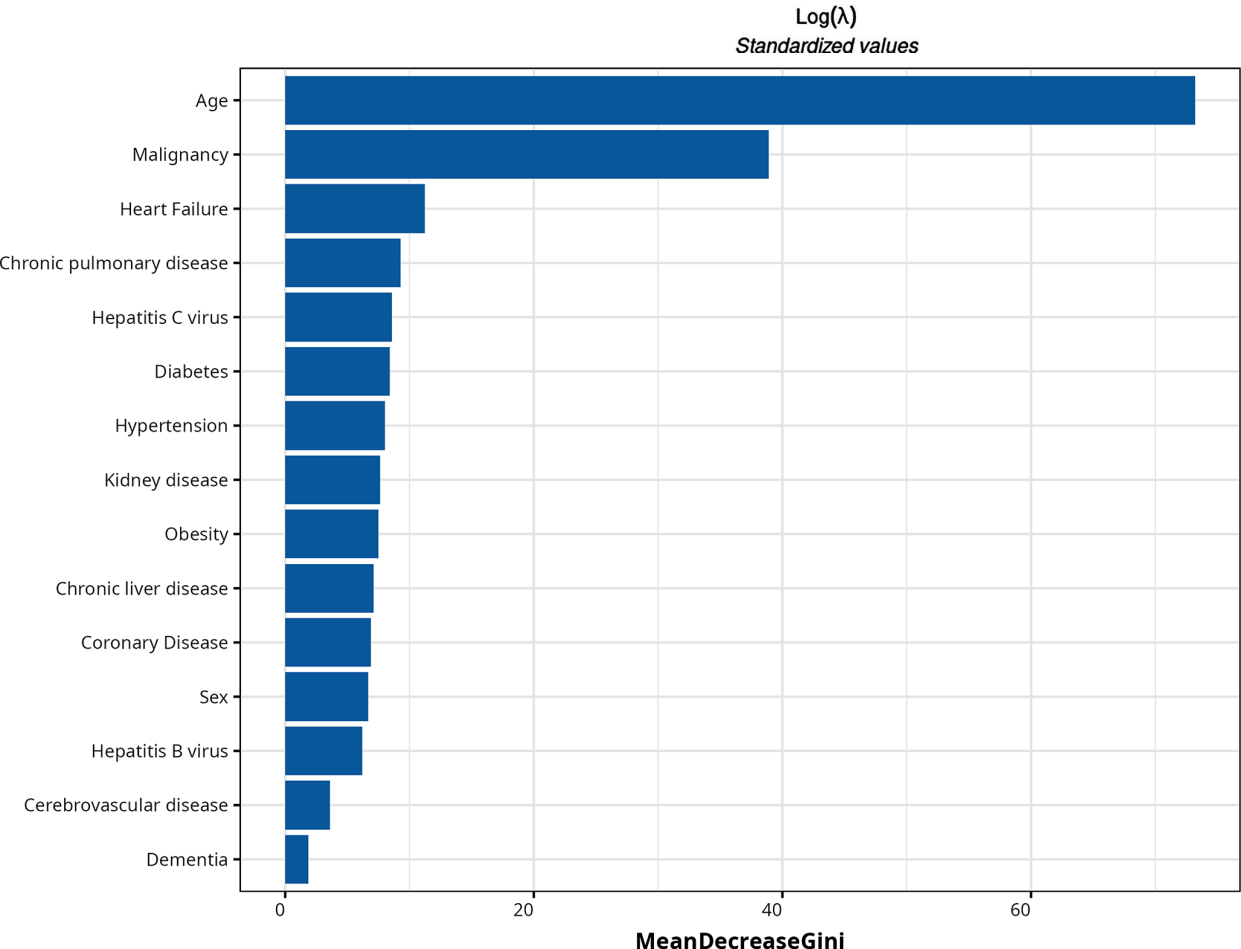
Importance of features based on the random forest algorithm

Finally, to improve the interpretation of the results, we used RF to rank variables in order of importance. The variable importance for the selected features can be examined visually to allow us to observe which were the most important for predicting the response variable. Using all variables, therefore, Fig. 4 shows the results provided by the RF algorithm, and the 15 features included are displayed. Age, malignancy, and acute heart failure were the most relevant variables, as identified by penalized logistic regression.

## Discussion

The main aim of our study was to determine the impact of COVID-19 in hospitalized PLWH. We found that PWLH with COVID-19 were at high risk for both hospitalization and mortality. Coinfection with HIV and SARS-CoV-2 was determined to be an independent risk factor for mortality when it was adjusted by age, sex, and other comorbidities.

More specifically, we found that hospitalized PLWH were younger than hospitalized non-PLWH. PLWH tended to have a lower prevalence of diabetes, obesity, and hypertension but a higher prevalence of chronic liver or pulmonary diseases. Malignancy was also higher in PLWH. This suggests that the demographic and clinical profiles among hospitalized PLWH are different than those in the general population. Below, we further discuss the relationship between these variables and the risk of mortality.

In a recent meta-analysis, the pooled prevalence of PLWH in European countries was 0.73% (95%CI 0.24–1.22), although there was risk of bias due to the small number of patients included [3]. Still, our results among hospitalized patients are similar to that prevalence. In the included European studies, the median age was 50, in line with the age of 54 in our study. In addition, in that meta-analysis, men represented 74% of all patients (the value was 75% in our study). Overall, our results are in line with those of Danwang et al. [3], with the exception of the mortality risk. Those authors did not find that HIV infection increases the likelihood of severe COVID-19 outcomes.

Our results are also in line with a large retrospective study performed in the UK [10] that found that PLWH were more likely to be men, with a median age of 48. That study also found that, after adjusting for age, sex, and other comorbidities, PLWH were at higher risk for death.

In our multivariate analyses, age and sex were risk factors for mortality, not only in the general population but also in PLWH [11, 12]. HIV infection was a risk factor for mortality once sex and age were included in adjusted analyses, as reported previously [13–17].

We found that the mortality rate was lower among PLWH in unadjusted univariate analyses (Table 1). However, when adjusted multivariate analyses were performed, we found that HIV infection was associated with increased risk of mortality (OR 1.25, 95%CI 1.14–1.37); that is, that PLWH are 25% more likely to die due to COVID-19 than non-PLWH. As noted, the meta-analysis by Danwang et al. [3] did not find evidence for a link between HIV infection and mortality risk in COVID-19 patients, although the authors identified two studies that suggested this association. Our results may seem controversial in light of this difference between univariate and multivariate outcomes with respect to the risk of mortality among PLWH. However, this phenomenon is well documented and can be explained by the fact that univariate analyses can miss some variables that are deemed relevant in multivariate analyses; for this reason, it can produce biased estimates of effects of other variables on the response [18].

Another controversy is that we also found that PLWH who were coinfected with SARS-CoV-2 were 75–110% more likely to be admitted to a hospital. Conditions such as HIV infection can be considered a risk factor, along with cardiovascular comorbidities, in patients with COVID-19. A preliminary report of a case-control study suggested that SARS-CoV-2 coinfection does not have an extraordinarily great impact on PLWH [19]. However, the authors emphasized limitations of their study and reported certain trends on severity and mortality that could be worse in PLWH than in non-PLWH. A later study suggested that PLWH are at increased risk for hospitalization [3] did not find an increased risk for adverse outcomes, including death. The authors referred to the role of immunodepression and immunovirological status in PLWH to explain their results. They hypothesized that the cytokine storm could be averted if immunodepression is present. They also proposed further research that would stratify immunovirological status, including CD4 + T lymphocytes counts and viral load, to identify patients that are most likely to present with severe forms of COVID-19. Bhaskaran et al. [10] demonstrated that HIV infection is a risk factor for mortality. They also stratified risk based on age and comorbidities.

Although well-controlled HIV infection has been associated with cardiovascular disease [17, 20, 21], the comorbidities analyzed in our cohort did not have a significant effect on mortality. Hypertension, obesity, chronic kidney disease, and coronary disease were risk factors in the general population but not in PLWH. We found that heart failure was associated with a higher risk of mortality in our cohort. PLWH may be at high cardiovascular risk, not only due to aging but also because anti-retroviral therapy (ART) may predispose PLWH to the development of cardiovascular diseases. Heart failure has been noted as an important comorbidity in PLWH, despite ART [22, 23].

It should be noted that we found not only a higher prevalence of malignancy among PLWH than among non-PLWH but also that cancer was a risk factor for mortality in the case of coinfection with SARS-CoV-2. In a recent multicenter study, Suarez et al. [24] investigated the relationship between malignancy and HIV in 17,978 PLWH in Spain. The authors found that mortality due to cancer was higher among PLWH than among the general population. Malignancy was split into several categories, including viral, nonviral, and non-AIDS-defining cancer, and they concluded that cancer was a risk factor for mortality in all categories analyzed.

Our study had several strengths. First, we used machine learning for data analyses, which gave us better insight into the results. In the first step, we used LASSO as an alternative to standard logistic regression. LASSO provides more parsimonious models through feature selection. It selects only a subset of relevant variables while irrelevant or noisy variables are dropped, with no effect on the accuracy of the resulting model. LASSO is simple and easy to understand. In the second step, to better interpret the effect of each variable in the resulting model, we used RF to rank the variables, ordered by their importance in the model. Overall, machine learning allows for better explanation of results, which can help clinicians obtain better insight from them. For these reasons, we believe that our results are robust and provide important implications.

Another strength of our study was its inclusion of almost all patients hospitalized due to COVID-19 in Spain within the observation period and therefore our ability to analyze almost all PLWH coinfected and hospitalized with SARS-CoV-2. Furthermore, this research depicts the situation of hospitalized PLWH in Spain over the first 3 years of the pandemic. In addition, we included demographic and clinical estimators to adjust the risk (sex, age, cardiovascular disease). However, we are aware that further studies with stratified CD4 + T lymphocytes counts, viral loads, and ART would help shed light on the risk for adverse outcomes in PLWH.

A major limitation of this study is the lack of information on immunovirological status and on the prevalence of ART. However, Spain reached the aim of the United Nations Programme on HIV, that is, the 90–90–90 target, in 2021 [25, 26], that is, 90% of all PLWH will know their HIV status, 90% of PLWH will receive ART, and 90% of people receiving ART will have viral suppression. It is therefore plausible to assume that at least 80% of inpatients have received antiretroviral therapy and exhibit viral suppression [26]. Bhaskaran et al. also had the limitation of not including data regarding ART, viral load, or CD4 + T lymphocytes status, but they did not consider that to have distorted their findings [10].

## Conclusions

PLWH have a greater chance of being hospitalized due to COVID-19 than non-PLWH. PLWH are 25% more likely to die if coinfected with SARS-CoV-2 than non-PLWH. Our results indicate that PLWH should be considered at risk for both hospitalization and adverse outcomes, including mortality. The effects of age, sex, and other comorbidities should also be considered as adjusting estimators because they can modify the clinical course of COVID-19 in PLWH.

## Data Availability

A contract signed with the Spanish Health Ministry, which provided the data set, prohibits the authors from providing their data to any other researcher. Furthermore, the authors must destroy the data upon the conclusion of their investigation. The data cannot be uploaded to any public repository.
